# Delayed Esophageal Perforation Diagnosed 12 Years After Anterior Cervical Diskectomy and Fusion: A Case Report and Review of Current Literature

**DOI:** 10.5435/JAAOSGlobal-D-22-00080

**Published:** 2022-10-13

**Authors:** Philip Zakko, Matthew Rontal, Daniel Park

**Affiliations:** From the Department of Orthopaedic Surgery (Dr. Zakko and Dr. Park) and the Department of Otolaryngology (Dr. Rontal), Beaumont Health, Royal Oak, MI.

## Abstract

Esophageal perforation associated with anterior cervical diskectomy and fusion (ACDF) is a rare but serious complication. ACDF-related esophageal perforations can be acute or delayed. Delayed perforations more than 10 years after ACDF are exceedingly rare. Here, a delayed esophageal perforation discovered 12 years after a three-level ACDF is presented. This case highlights two main points. First, all diverticula after an ACDF warrant close clinical monitoring. Second, routine follow-up should be performed for patients with screw pullout to assist in early diagnosis of delayed esophageal perforation.

Anterior cervical diskectomy and fusion (ACDF) is one of the most common spinal surgeries in the United States.^1^ Outcomes are often favorable with surgical success ranging from 85% to 95%.^2^ However, there is risk of injury to nearby anatomic structures, such as the esophagus, leading to serious complications.

Esophageal perforation associated with ACDF is rare with rates reported between 0.02% and 1.52%.^3^ Perforations can lead to fistulas, abscesses, osteomyelitis, mediastinitis, and sepsis. With timely intervention, mortality is as high as 20%.^4^ With delays in treatment, mortality approaches 50%.^5,6^ The clinical presentation of esophageal perforation is variable. Thus, diagnosis is challenging. There is no clear consensus on the management of esophageal perforation after ACDF.

Acute esophageal perforation within the first 30 days of surgery is most commonly an intraoperative injury from sharp instrumentation, burr, electrocautery, or inadvertent retractor placement.^3^ Delayed esophageal perforation is most commonly due to hardware failure and chronic erosion.^7,8^ Acute perforations are more common than delayed, and perforations more than 10 years after ACDF are exceedingly rare.^7,8,9,10^

We present a delayed esophageal perforation discovered 12 years after a three-level ACDF. This case suggests that delayed esophageal perforation should remain on a clinician's radar years after the index procedure.

## Case Report

### Clinical Presentation

**Figure 1:**
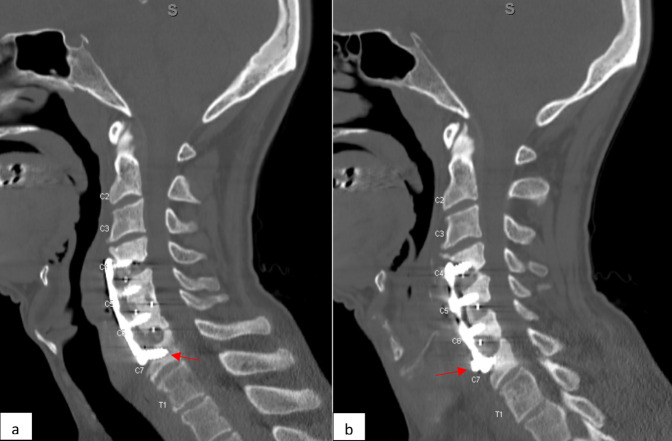
CT of the cervical spine 7 years after ACDF demonstrating (**A**) nonunion of C4-5 and C6-7 with a lucency around the left C7 screw and (**B**) 3-mm pullout of right C7 screw. ACDF = anterior cervical diskectomy and fusion

**Figure 2:**
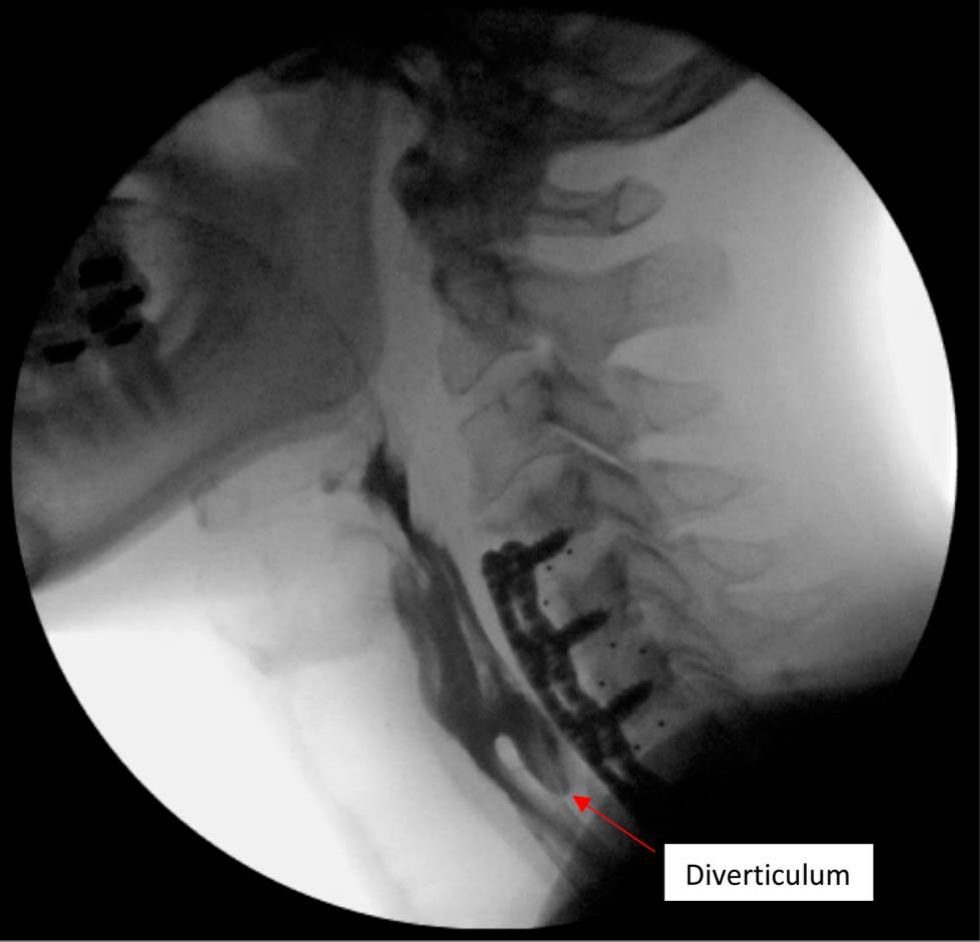
Barium swallow showing 10 years after index ACDF diagnosed as a Zenker diverticulum. However, in the setting of anterior cervical hardware, this is most consistent with an ACDF-related diverticulum. ACDF = anterior cervical diskectomy and fusion

A 51-year-old man presented to our outpatient orthopaedic clinic with a few years of progressive neck pain and dysphagia. He reported sharp, stabbing, and burning pain radiating to his right trapezius. He was evaluated for these symptoms 2 years prior but received no treatment. He reported a current visual analog scale neck pain of 5.36, a moderate Bazaz dysphagia score with difficulty swallowing many solids but not liquids, and an EAT-10 of 26. Surgical history was notable for a C4-7 ACDF performed 12 years prior by an outside surgeon for symptoms which improved after surgery. He reported no surgical wound issues or hospitalizations for sepsis from date of surgery to present. Outside records were notable for a CT of the cervical spine 2 years prior, demonstrating nonunion of C4-5 and C6-7 with lucency around the left C7 screw and 3-mm pullout of the right C7 screw (Figure 1). A barium swallow performed around the same time was diagnosed as a Zenker diverticulum (Figure 2). Neither the loose screw nor the diverticulum received further workup or intervention.

**Figure 3:**
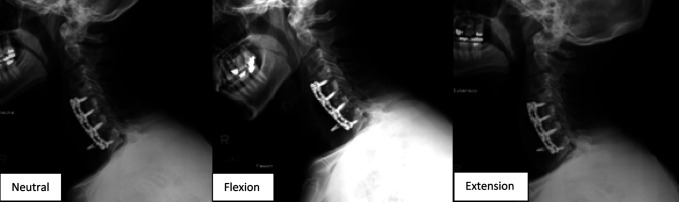
Cervical spine x-rays 12 years after ACDF demonstrating a completely loose and 180° rotated right C7 screw, along with gas anterior to the C4-7 ACDF plate. ACDF = anterior cervical diskectomy and fusion

**Figure 4:**
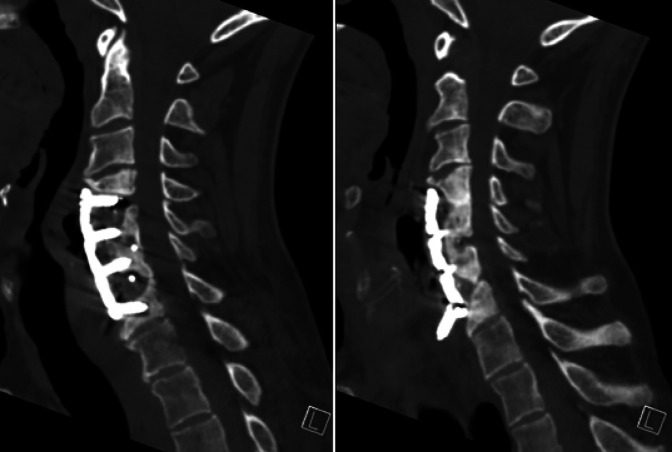
CT of the cervical spine 12 years after ACDF demonstrating pseudoarthrosis at C4-5 and C6-7, erosive changes to the vertebral bodies of C4-5 and C6-7 with loss of bone directly posterior to the plate, loosening around the left C7 screw (left image), anterior displacement of the right C7 screw (right image), and fluid/air anterior to the surgical plate. ACDF = anterior cervical diskectomy and fusion

In our clinic, cervical x-rays revealed a completely loose and 180° rotated right C7 screw (Figure 3). A CT showed pseudarthrosis and erosive changes to the vertebral bodies of C4-5 and C6-7 with loss of bone directly posterior to the plate, anterior displacement of the right C7 screw, and fluid/air anterior to the plate (Figure 4). The patient was diagnosed with suspected chronic esophageal perforation with hardware failure and pseudarthrosis.

### Surgical Intervention

A joint procedure between a fellowship-trained orthopaedic spine surgeon (D.P.) and a fellowship-trained otolaryngologist (M.R.) was performed. With the patient supine, an anterior cervical skin incision was made on the right side in correspondence with the incision from the index procedure. The right C7 screw was extracted from the plate and removed. Gross purulence and food debris were evident around the plate. Cultures were obtained. The plate was removed. The C4-5 and C6-7 interbody cages were loose. The C5 and C7 vertebral bodies were eroded from infection, leaving defects of 14 mm at the C4-5 and C6-7 disk spaces. After copious irrigation and débridement, titanium cages were placed and buttressed by plates across the C4-5 and C6-7 spaces.

Next, the esophagus was explored, and a 4-cm vertical defect was identified. Consideration was given to a regional flap; however, reasonable laxity of the esophagus allowed for primary repair. After repair, a C4-7 posterior spinal fusion with lateral mass screws was performed the same day.

### Flap Coverage

**Figure 5:**
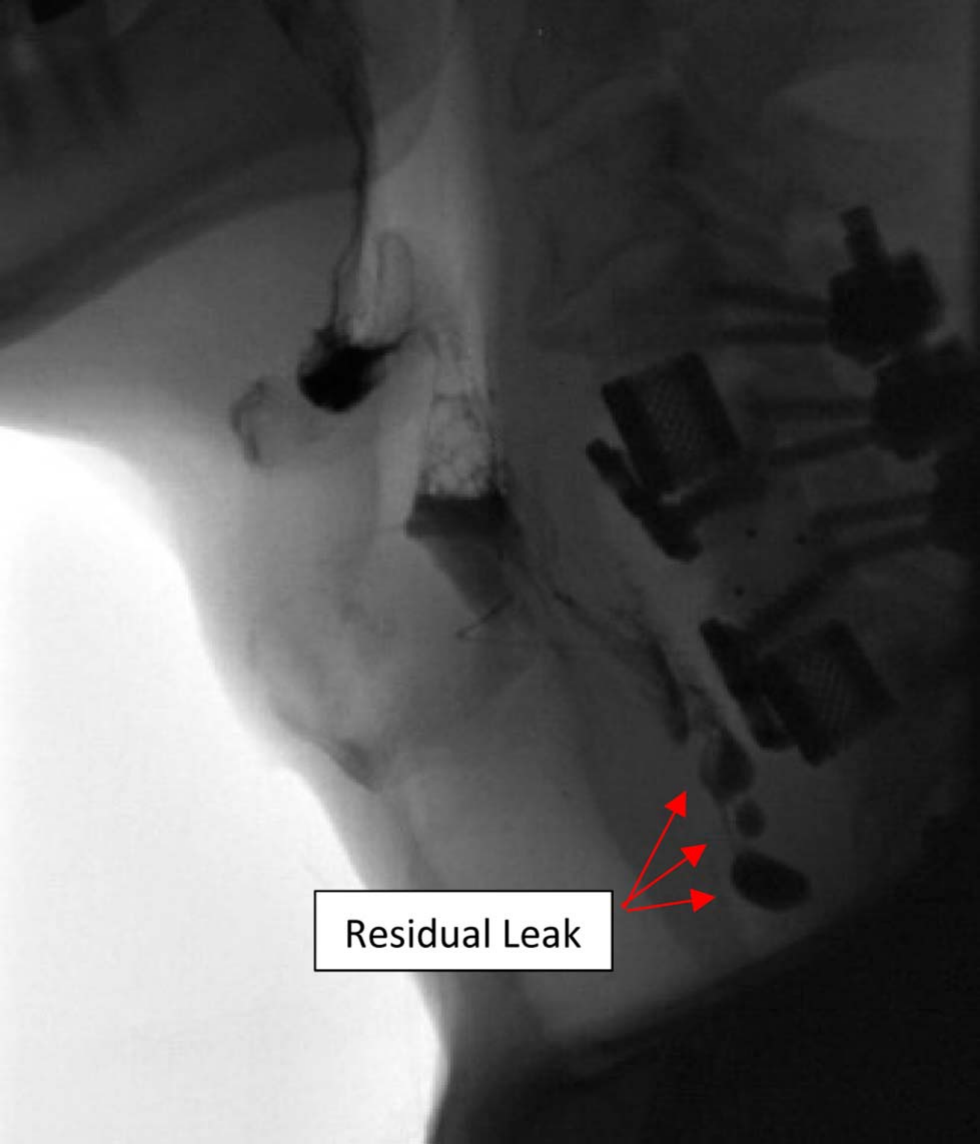
Barium swallow performed 10 days postoperatively showing residual esophageal leak.

**Figure 6:**
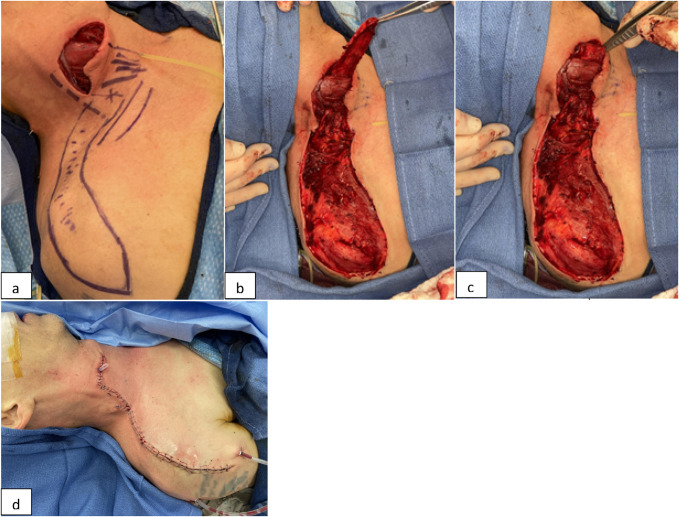
Pictures showing esophageal reconstruction with a SCAIF. **A**, The triangle between the external jugular vein, clavicle, and sternocleidomastoid muscle was marked, and the pulse from the transverse cervical artery was identified. This pulse was followed laterally into the deltoid region as the supraclavicular branch of the transverse cervical artery. **B**, An 18-cm long and 5-cm wide flap was harvested and demonstrated good doppler signals. **C**, The flap was then de-epithelialized and rotated into the paraoesophageal region through a tunnel under the sternocleidomastoid muscle to cover the primary esophageal repair, the great vessels, and the spine. **D**, The surgical site was closed with drains placed into the paraoesophageal region and the flap donor site. SCAIF = supraclavicular artery island flap

The patient initially did well with improvement in his neck pain. However, over days, he progressively lost the ability to tolerate tube feeds. On postoperative day 10, an esophagram showed a persistent esophageal leak (Figure 5). Thus, the decision was made for an esophageal reconstruction with a supraclavicular artery island flap (SCAIF) (Figure 6).

### Postoperative Course

After surgery, the patient remained in a c-collar for 2 weeks, had a percutaneous endoscopic gastrostomy tube placed, and was kept nil per oral for 3 months. Surgical cultures grew *Lactobacillus gasseri*, *Rothia mucilaginosa*, and *Candida albicans* which were treated with 1 month of IV ampicillin/sulbactam through a peripherally inserted central catheter and fluconazole through his percutaneous endoscopic gastrostomy tube.

**Figure 7:**
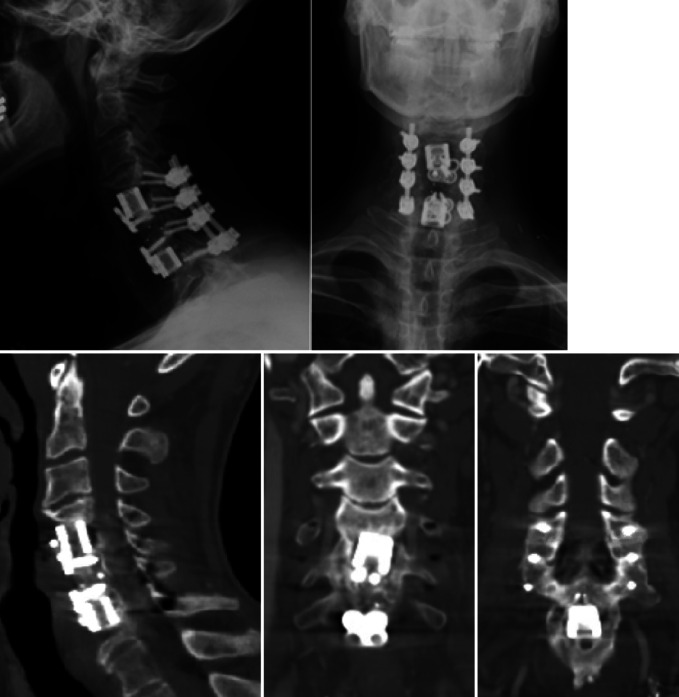
Cervical spine radiograph and CT performed 1 year postoperatively demonstrating C4-7 fusion with stable constructs and no signs of loosening or hardware failure.

**Figure 8:**
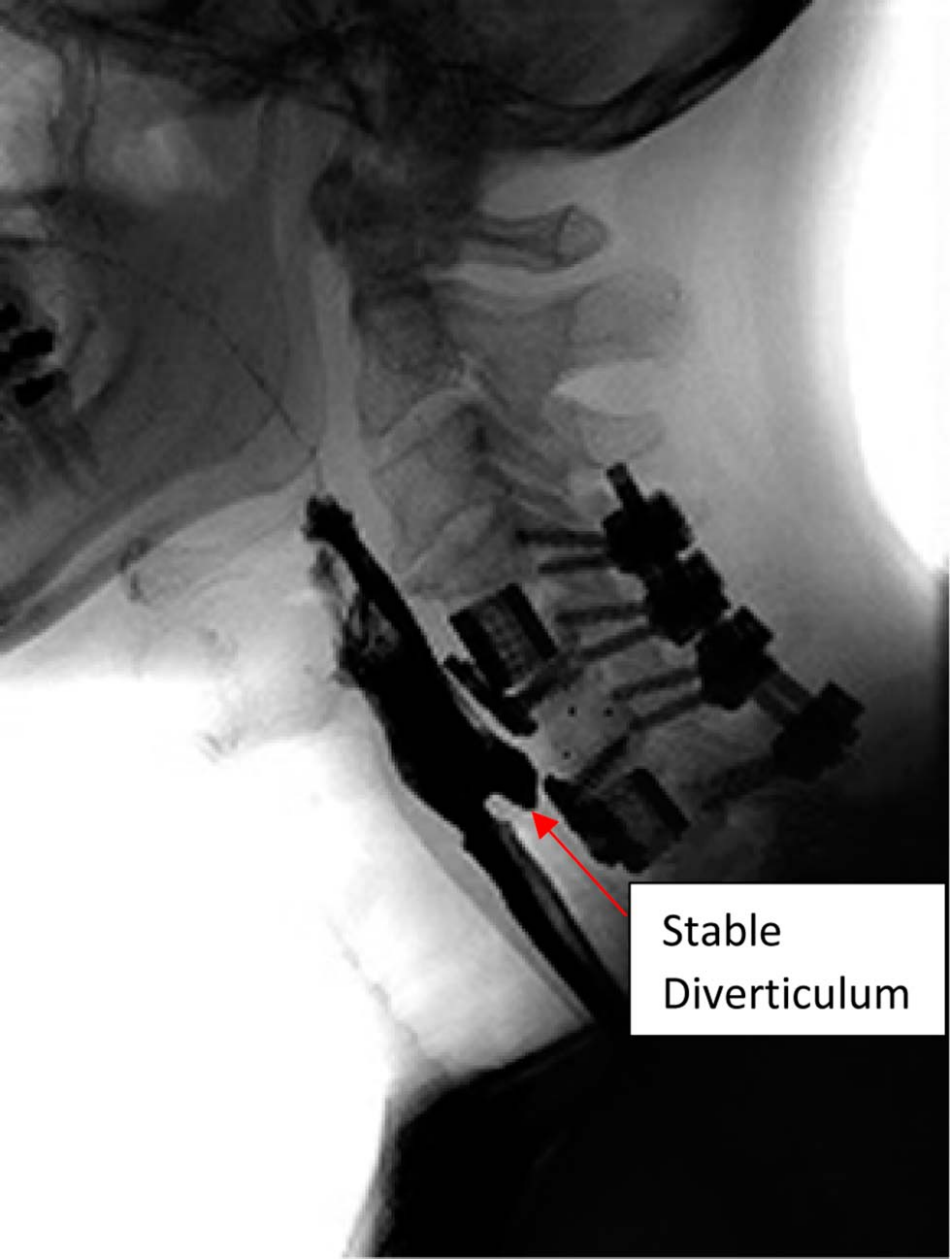
Barium swallow 1 year postoperatively showing stable diverticulum with no evidence of esophageal leak.

Most recently, he continues to have a moderate Bazaz with occasional swallowing difficulties to solids such as meats but no difficulty with liquids. He has a slight improvement in his postoperative EAT-10 of 18. He also reports improvement in his postoperative visual analog scale neck pain of 3.5. His neck disability index is 56, PROMIS physical score is 31, and EQ-5D is 0.797. His C-reactive protein (6.3 mg/L) and erythrocyte sedimentation rate (15 mm/hr) both normalized at 1 month after surgery. He is currently off all antibiotics. At 1 year follow-up, imaging confirmed C4-7 fusion with stable hardware (Figure 7). Barium swallow showed no recurrent esophageal leaks (Figure 8).

## Discussion

This case highlights a delayed esophageal perforation discovered 12 years after the index ACDF. This is an exceedingly rare complication after ACDF with only four other known reports occurring more than 10 years after surgery.^7,8,9,10^ Early recognition and treatment are paramount to reduce morbidity and mortality; however, diagnosis can be difficult, especially in delayed presentations.

Screw loosening and hardware failure are often causes of delayed esophageal perforation. In a study by Tasiou et al,^11^ the reported incidence of screw pullout after ACDF was 0.9%. In a systematic review of esophageal injury after ACDF, 41% of esophageal perforations were due to hardware failure (24% from screw and/or plate loosening) and 31% due to chronic erosion by hardware.^3^ Although the occurrence of screw pullout with associated esophageal perforation is relatively rare, early detection and management could prevent complications.

The management of screw loosening varies. There are reports of conservative management in asymptomatic patients with screw pullout but no plate displacement.^12^ For screw loosening of 2 to 5 mm, Ning et al found that conservative treatment with Philadelphia collar immobilization for 3 months was successful with bony fusion in all patients.^13^ Lowery also found that screw loosening of 5 mm or less does not increase risk of injury to nearby anatomic structures; however, loosening of 5 mm or more can increase risk of esophageal perforation.^14^ Before presentation at our institution, our patient had a CT scan demonstrating 3-mm pullout of the right C7 screw. Therefore, we recommend routine follow-up radiographs for all symptomatic and asymptomatic patients who demonstrate any degree of pullout, especially in the setting of a nonunion.

Furthermore, the preoperative CT showed bone loss directly posterior to the ACDF plate with erosive changes around the cages of C4-5 and C6-7. Especially in a patient with increasing neck pain and dysphagia, these findings increase suspicion for chronic infection with osteomyelitis potentially in the setting of an esophageal leak. Thus, suspicion for infection should be raised in any patient with increasing bone loss seen on imaging after an ACDF.

In addition, this patient was diagnosed with a Zenker diverticulum years after his index ACDF. Zenker diverticulum, a false diverticulum involving the esophageal mucosa and submucosa, is a very rare complication after anterior spine surgery.^15,16,17,18,19^ Alternatively, ACDF-related diverticulum, a true diverticulum involving the esophageal mucosa, submucosa, and muscular layers, is thought to be due to dense scar tissue adhering the esophagus to the vertebral body and surgical hardware.^19,20^ As a true diverticulum, there is increased risk of esophageal perforation compared with Zenker. However, ACDF-related diverticulum can be easily misdiagnosed as a Zenker diverticulum.^20,21^ For these reasons, any diverticulum after ACDF should be considered an ACDF-related diverticulum until proven otherwise to allow prompt treatment and prevent complications such as perforation.

Regarding treatment, both nonoperative and operative management for perforations are used. The criteria for nonoperative management include early diagnosis or delayed diagnosis with contained leak, perforation not in the abdomen, contained in the mediastinum, not involving neoplasm or obstruction, absence of sepsis, presence of experienced thoracic surgeon, and contrast imaging in the hospital.^22,23^ Nonoperative management involves supportive care with nil per oral status and IV antibiotics.^3,24^ Although nonoperative management can be successful in the appropriate setting, most esophageal perforations require surgical intervention.

Rueth et al^25^ devised an algorithm for management of esophageal perforation after anterior cervical spine surgery. For intraoperative perforation, they recommend primary repair. For suspected perforation after surgery, they recommend diagnosis with clinical examination, esophagoscopy, and contrast swallow studies. If these studies suggest perforation, surgical débridement with primary repair should be performed. If repeat studies in 2 to 3 weeks show residual leak, surgical revision with or without flap coverage should be performed.

When flap coverage is necessary, it is essential that the flap is adequately vascularized. With esophageal perforations, the surrounding soft tissue is often compromised with infection, scar, and poor wound healing. Thus, local flaps, such as sternocleidomastoid and longus colli, are often limited in viability leading the surgeon to choose from other vascularized flaps including pleural, omental, pedicled pectoralis major, free radial forearm, and omental flaps.^26^

In this case, we elected for SCAIF coverage. Consideration was initially given to radial forearm free flap reconstruction, but the patient's Allen test did not demonstrate an intact palmar arch. A pectoralis major flap was considered; however, the patient worked as an auto mechanic, and the donor site morbidity would not be acceptable. An infrahyoid flap would not be reliable because infection and debris had infiltrated the strap muscles.

There is also controversy regarding use of hardware during the primary surgical procedure in patients with cervical osteomyelitis. Similar to a periprosthetic joint infection, infection in the setting of spinal instrumentation leads to presumed biofilm formation.^27^ As such, removal of all hardware in a primary procedure is thought to be essential with return to operating room for hardware placement at a later date after eradication of infection.^25^ In cases of an unstable spine after primary hardware removal, various temporary stabilizing measures have been described including cervical external fixation, posterior spinal fusion, and rigid cervical collar application.^25,28,29^ Although there is a lack of literature regarding osteomyelitis after ACDF, newer literature suggests that use of hardware during the primary surgical procedure for cervical osteomyelitis may allow for appropriate eradication of infection. In a systematic review of 239 patient across 24 studies treated for cervical spine osteomyelitis, Wang et al found that primary placement of instrumentation during active infection did not increase the rates of hardware failure or wound complications compared with those of elective cervical spine procedures.^30^ Aryan et al^31^ further found that corpectomy followed by placement of titanium cages in the setting of osteomyelitis did not lead to recurrent hardware infections. Titanium implants are shown to have reduced propensity for biofilm formation compared with polyether-ether-ketone and stainless-steel implants, thus supporting its potential safety.^27^

In this case, given the amount of osteomyelitis and degree of bony removal required, our patient would have had significant spinal instability without hardware stabilization. Temporary external fixation was considered at the index procedure; however, in consultation with ENT it was believed that we had adequate débridement and repair to allow a one-stage procedure. Unfortunately, there was found to be residual leak requiring a secondary procedure and SCAIF. Regardless, even with placement of stabilizing hardware, the patient adequately eradicated his infection as suggested by his lack of wound complications, normal postoperative C-reactive protein and erythrocyte sedimentation rate, and lack of systemic signs of infection.

## Conclusion

This patient had an ACDF-related diverticulum with chronic delayed esophageal perforation. Earlier identification and surgical intervention may have alleviated the need for extensive esophageal flap coverage and anterior cervical spine revision surgery. This case highlights two main points. First, all diverticula after an ACDF warrant close clinical monitoring. Second, routine follow-up should be performed for patients with screw pullout to assist in early diagnosis of delayed esophageal perforation.

## References

[1] Epstein NE: A review of complication rates for anterior cervical diskectomy and fusion (ACDF). Surg Neurol Int 2019;10:100. 31528438 10.25259/SNI-191-2019 PMC6744804

[2] Buttermann GR: Anterior cervical discectomy and fusion Outcomes over 10 years: A prospective study. Spine (Phila Pa 1976) 2018;43:207-214. 28604488 10.1097/BRS.0000000000002273

[3] Halani SH Baum GR Riley JP : Esophageal perforation after anterior cervical spine surgery: A systematic review of the literature. J Neurosurg Spine 2016;25:285-291. 27081708 10.3171/2016.1.SPINE15898

[4] Orlando ER Caroli E Ferrante L: Management of the cervical esophagus and hypofarinx perforations complicating anterior cervical spine surgery. Spine 2003;28:E290-E295. 12897507 10.1097/01.BRS.0000087093.89889.0A

[5] Bladergroen MR Lowe JE Postlethwait RW: Diagnosis and recommended management of esophageal perforation and rupture. Ann Thorac Surg 1986;42:235-239. 3753071 10.1016/s0003-4975(10)62725-7

[6] Pompili A Canitano S Caroli F : Asymptomatic esophageal perforation caused by late screw migration after anterior cervical plating: Report of a case and review of relevant literature. Spine 2002;27:E499-E502. 12461406 10.1097/00007632-200212010-00016

[7] Almre I Asser A Laisaar T: Pharyngoesophageal diverticulum perforation 18 years after anterior cervical fixation. Interact Cardiovasc Thorac Surg 2014;18:240-241. 24246672 10.1093/icvts/ivt421 PMC3895056

[8] Park M-K Cho D-C Bang W-S Kim K-T Sung J-K: Recurrent esophageal perforation after anterior cervical spine surgery: Case report. Eur Spine J 2018;27(suppl 3):515-519. 29500543 10.1007/s00586-018-5540-1

[9] Thomas JP Finch R: Esophageal erosion. A complication of acrylic fixation in anterior cervical fusion. Spine (Phila Pa 1976) 1991;16:1238-1240. 1754945

[10] Gibson AW Gobillot TA Bass DI Zakarevicius Z Rizvi ZH Ravanpay AC: Case of esophageal perforation and repair with a supraclavicular artery island fascial flap 15 years after anterior spine surgery. World Neurosurg 2020;143:102-107. 32730966 10.1016/j.wneu.2020.07.151

[11] Tasiou A Giannis T Brotis AG : Anterior cervical spine surgery-associated complications in a retrospective case-control study. J Spine Surg 2017;3:444-459. 29057356 10.21037/jss.2017.08.03 PMC5637201

[12] Geyer TE Foy MA: Oral extrusion of a screw after anterior cervical spine plating. Spine (Phila Pa 1976) 2001;26:1814-1816. 11493857 10.1097/00007632-200108150-00019

[13] Ning X Wen Y Xiao-Jian Y : Anterior cervical locking plate-related complications; prevention and treatment recommendations. Int Orthop 2008;32:649-655. 17497150 10.1007/s00264-007-0369-y PMC2551717

[14] Lowery GL McDonough RF: The significance of hardware failure in anterior cervical plate fixation. Patients with 2- to 7-year follow-up. Spine (Phila Pa 1976) 1998;23:181-186. 9474723 10.1097/00007632-199801150-00006

[15] Ba AM LoTempio MM Wang MB: Pharyngeal diverticulum as a sequela of anterior cervical fusion. Am J Otolaryngol 2006;27:295-297. 16798413 10.1016/j.amjoto.2005.11.009

[16] Summers LE Gump WC Tayag EC Richardson DE: Zenker diverticulum: A rare complication after anterior cervical fusion. J Spinal Disord Tech 2007;20:172-175. 17414989 10.1097/BSD.0b013e31802c1474

[17] Joanes V Belinchón J: Pharyngoesophageal diverticulum following cervical corpectomy and plating. Case report. J Neurosurg Spine 2008;9:258-260. 18928221 10.3171/SPI/2008/9/9/258

[18] Goffart Y Moreau P Lenelle J Boverie J: Traction diverticulum of the hypopharynx following anterior cervical spine surgery. Case report and review. Ann Otol Rhinol Laryngol 1991;100:852-855. 1952654 10.1177/000348949110001012

[19] Tian H Yuan W Johnson JS Chen H Chen D: Pharyngoesophageal diverticulum: A delayed complication of anterior cervical spine surgery. Eur Spine J 2011;20(suppl 2):S211-S216. 20927556 10.1007/s00586-010-1579-3 PMC3111524

[20] Park JM Kim CW Kim DH: Acquired pharyngeal diverticulum after anterior cervical fusion operation misdiagnosed as typical Zenker diverticulum. Korean J Thorac Cardiovasc Surg 2016;49:309-312. 27525244 10.5090/kjtcs.2016.49.4.309 PMC4981237

[21] Watemberg S Landau O Avrahami R: Zenker's diverticulum: Reappraisal. Am J Gastroenterol 1996;91:1494-1498. 8759648

[22] Cameron JL Kieffer RF Hendrix TR Mehigan DG Baker RR: Selective nonoperative management of contained intrathoracic esophageal disruptions. Ann Thorac Surg 1979;27:404-408. 110275 10.1016/s0003-4975(10)63335-8

[23] AltorjAy A Kiss J Vörös A : Nonoperative management of esophageal perforations. Is it justified? Ann Surg 1997;225:415-421. 9114801 10.1097/00000658-199704000-00011 PMC1190750

[24] Kaman L Iqbal J Kundil B Kochhar R: Management of esophageal perforation in adults. Gastroenterol Res 2010;3:235-244. 10.4021/gr263w PMC5139851 27942303

[25] Rueth N Shaw D Groth S : Management of cervical esophageal injury after spinal surgery. Ann Thorac Surg 2010;90:1128-1133. 20868802 10.1016/j.athoracsur.2010.06.045

[26] Hanwright PJ Purnell CA Dumanian GA: Flap reconstruction for esophageal perforation complicating anterior cervical spinal fusion: An 18-year experience. Plast Reconstr Surg Glob Open 2015;3:e400. 26090290 10.1097/GOX.0000000000000350 PMC4457263

[27] Kasliwal MK Tan LA Traynelis VC: Infection with spinal instrumentation: Review of pathogenesis, diagnosis, prevention, and management. Surg Neurol Int 2013;4(suppl 5):S392-S403. 24340238 10.4103/2152-7806.120783 PMC3841941

[28] Dakwar E Uribe JS Padhya TA Vale FL: Management of delayed esophageal perforations after anterior cervical spinal surgery. J Neurosurg Spine 2009;11:320-325. 19769513 10.3171/2009.3.SPINE08522

[29] Ardon H Van Calenbergh F Van Raemdonck D : Oesophageal perforation after anterior cervical surgery: Management in four patients. Acta Neurochir (Wien) 2009;151:297-302. 19255711 10.1007/s00701-009-0241-5

[30] Wang AJ Huang KT Smith TR : Cervical spine osteomyelitis: A systematic review of instrumented fusion in the modern era. World Neurosurg 2018;120:e562-e572. 30165226 10.1016/j.wneu.2018.08.129

[31] Aryan HE Lu DC Acosta FL Ames CP: Corpectomy followed by the placement of instrumentation with titanium cages and recombinant human bone morphogenetic protein-2 for vertebral osteomyelitis. J Neurosurg Spine 2007;6:23-30. 17233287 10.3171/spi.2007.6.1.23

